# Is there an association between mild cognitive impairment and dietary pattern in chinese elderly? Results from a cross-sectional population study

**DOI:** 10.1186/1471-2458-10-595

**Published:** 2010-10-08

**Authors:** Ziqi Wang, Birong Dong, Guo Zeng, Jun Li, Wenlei Wang, Binyou Wang, Qiyuan Yuan

**Affiliations:** 1 Department of Geriatrics West China Hospital, West China School of Medicine, Sichuan University, China; 2 Department of Nutrition and Food Hygiene, West China School of Publish Health, Sichuan University, China

## Abstract

**Background:**

Diet has an impact on cognitive function in most prior studies but its association with Mild Cognitive Impairment (MCI) in Chinese nonagenarians and centenarians has not been explored.

**Methods:**

870 elder dujiangyan residents aged 90 years or more in 2005 census were investigated at community halls or at home. They underwent the Mini-Mental State Examination (MMSE) for assessment of cognitive function and replied to our questionnaire comprised of 12 food items and other risk factors. MCI was defined by two steps: first, subjects with post-stroke disease, Alzheimer's disease or Parkinson's disease and MMSE< 18 were excluded; and then subjects were categorized as MCI (MMSE scores between 19 and 24) and normal (MMSE scores between 25 and 30). Logistic regression models were used to analyze the association between diet and the prevalence of MCI. The model was adjusted for gender, ages, systolic blood pressure, diastolic blood pressure, body mass index, fasting plasma glucose, total cholesterol, triglycerides, high-density lipoprotein cholesterol and low-density lipoprotein cholesterol, smoking habits, alcohol and tea consumption, educational levels and exercise in baseline dietary assessment.

**Results:**

364 elderly finally included, 108 (38.71%) men and 171 (61.29%) women of whom were classified as MCI. A significant correlation between MCI and normal in legume was observed (OR, 0.84; 95%CI, 0.72-0.97), and also in animal oil (any oil that obtained from animal substances) (OR, 0.93; 95%CI, 0.88-0.98). There was no statistical difference of other food items between normal and MCI.

**Conclusions:**

Among Chinese nonagenarians and centenarians, we found there were significant associations between inadequate intake of legume and animal oil and the prevalence of MCI. No significant correlation between other food items and the prevalence of MCI were demonstrated in this study.

## Background

Mild cognitive impairment (MCI) is a subjective complaint of memory impairment with objective memory impairment adjusted for age and education in the absence of dementia [1]. It ranges from normal aging to dementia in a species of the transitional stage of cognitive impairment [2], and may be early signs of Alzheimer's disease (AD). In 2001, at the "Current Concepts in MCI Conference," a definition of MCI that more broadly encompassed the clinical heterogeneity of MCI patients beyond memory impairment was proposed. Three subsets of MCI were proposed: 1) amnestic-MCI; 2) multiple domains, slightly-impaired-MCI; and 3) single, non-memory-domain-MCI. Patients with MCI may show evidence of vascular disease; movement disorders without diagnosed Parkinson disease; or neuropsychiatric disorders. In accordance with the clinical categorization of dementia, MCI subtypes based on etiology have been proposed. But it still lacks standardized diagnostic criteria of MCI [3]. Dementia is the advanced stage of untreated or mistreated mild cognitive impairment, and early diagnosis and intervention of mild cognitive impairment could postpone or prevent the onset of subsequent dementia [4]. As a curative treatment is currently impossible, current studies are major in behavioural or pharmacological preventive interventions. Among behavioural approaches, diet may play an important role in the causation and prevention of AD. However, epidemiological data on diet and AD have been conflicting [5,6]. Moreover, there is paucity of research with regard to the effect of dietary factors on the prevalence of MCI in Chinese old people (especially in Nonagenarians and Centenarians).

Recently, a study demonstrated that higher adherence to the MeDi (a diet characterized by high intake of fish, vegetables, legumes, fruits, cereals, unsaturated fatty acids (mostly in the form of olive oil), low intake of dairy products, meat, saturated fatty acids and a regular but moderate amount of ethanol) [7] is associated with a trend for reduced risk for developing MCI and with reduced risk for MCI conversion to AD [8]. Another study reported AD factor may be labelled as the low vegetable, high fat and sugar diet patter [9]. Nevertheless, potential associations between the Chinese' diet and MCI (among nonagenarians and centenarians) have not been explored. The primary aim of this paper is to investigate the association between Chinese' dietary pattern and MCI, using data from the Project of Longevity and Aging in Dujiangyan (PLAD).

## Methods

### Participants and Study Design

The data were collected by Project of Longevity and Aging in Dujiangyan (PLAD). The PLAD was initiated in April 2005 (and ended in Sep 2009) and aimed at investigating the relationship between environments, life-style, genetic, longevity and age-related diseases. Dujiangyan (located in Sichuan province and outside Chengdu city) inhabits 2,311,709 persons and 870 very-elderly-persons aged 90 years or more. The design of this study was questionnaire-based and cross-sectional in April 2005. The results of the questionnaire and health examination were filled in the standard form. Overall, 21 men and 26 women were not eligible for the study because they had already died or moved away from the area. 262 men and 561 women were interviewed, and we excluded subjects who suffered from post-stroke disease, Alzheimer's disease or Parkinson's disease (23 men and 31 women), or did not complete the MMSE test (8 men and 15 women), or the MMSE score ≤18 (71 men and 311 women). Finally, 364 participants (160 men and 204 women) were included in our study and their data were analyzed. The PLAD was approved by the Research Ethics Committee of the Sichuan University and written informed consent was obtained from all participants (also from their legal proxies).

### Data Collection

Data was obtained by trained personnel, who interviewed all the participants face to face. Participants were underwent a standardized physical examination, anthropometric measurements and a 12-lead electrocardiogram, based on the prepared questionnaire for the medical record. The following information was collected: age, gender, education level, the MMSE score, dietary habits, weight (kilograms), height (centimetres), tea drinking, alcohol consumption, smoking history and other items. Body mass index (BMI) was calculated as body weight in kilograms divided by height in metres squared. Venous blood samples were collected after an overnight fast (at least 8 h) for measurement of plasma glucose, plasma lipids and other biochemistry indicators. Sitting or recumbent position, right arm blood pressure (BP) was measured twice to the nearest 2 mm Hg using a standard mercury sphygmomanometer (phases I and V of Korotkoff) by trained nurses or physicians. The mean value of two measurements was used to calculate systolic BP (SBP) and diastolic BP (DBP), and the SBP and DBP were calculated as the mean of right and left arm values in exceptional subjects.

### Assessment of Cognitive Function

Cognitive function was assessed by the Mini-mental state examination (MMSE) test which includes 30-items component of orientation, attention, calculation, language and recall. The conventional cut-off score of cognitive impairment was defined as a score below 24 on the MMSE (sensitivity 80-90%, specificity 80-100%) [10]. But more and more studies pay close attention to many other factors (such as education and ages) affecting on the MMSE score. In women, at age 75, MMSE score ranged from 21 (10th percentile) to 29 (90th percentile). At age 95, the range was 10 (10th percentile) to 27 (90th percentile [11]. Cut-off points for old-old Chinese individuals (age 75 and older) with 0 to 6 years of education, the cut-off were 18/19 (sensitivity 94%, specificity 92%). For old-old Chinese individuals with more than 6 years of education, the cut-off was 22/23 (sensitivity 100%, specificity 88%) [12]. Previous study categorized subjects as follows: cognitive impairment (scores between 0 and 18), mild cognitive impairment (scores between 19 and 24) and normal (scores between 25 and 30) [10].

To decrease methodological bias and assure methodological reliability, the diagnosis of MCI was achieved as: mild cognitive impairment (scores between 19 and 24) and normal (scores between 25 and 30). Subjects were divided into 2 groups; those two groups were compared with each other on baseline characteristics and dietary patterns.

### Dietary Pattern Study Design

All participants filled out the questionnaire about the frequencies of each food item and the frequency units (day, week, month, year or never) based on12 food categories (grain (or cereals), vegetables, fruit, poultry, meat (pork, beef and mutton), eggs, fish and shrimp, milk and milk products, legumes, animal oil, plant oil and nuts. The 12 foods were based on the Chinese Food Guide Pagoda which is extensively used for dietary guidelines for Chinese residents. For easy to compute, each food category frequency was converted into times per week (times/week).

### Statistical Methods

SPSS16.0 was used in our analysis. All clinical continuous variables are presented as means ± standard deviation (M ± s.d.). Gender, educational level, current smoker, alcohol consumption and alcohol, smoking history and so on, for each group, were presented as category variables. Significance testing of the difference between the two groups was analysed using Independent-Samples t test for continuous variables and Chi-square or Fisher's exact test for category variables. Binary logistic analyses were performed to evaluate the association between potential dietary pattern risk factors and MCI. We calculated the 95% confidence interval (CI) for each odds ratio. P-value < 0.05 was considered to be statistically significant, and all of the P-values are two sides.

## Results

### Baseline Characteristics and Prevalence of Dietary Pattern and MCI

Among the 364 volunteers, mean age was 93.02 years (s.d. 3.01 years, range from 90 to105 years) and 204 (56.00%) were women, including 15 centenarians. 90% of subjects lived in the countryside. The mean cognitive function score for the old population was 22.52 (SD 2.75, range 19-30). In the oldest group, the total prevalence rate of mild cognitive impairment was 76.6%, the prevalence rate among males was 67.5% and among females it was 83.8%. Women had significantly higher prevalence of MCI than men (171/204 vs. 108/160 p < 0.001). Subjects without MCI had significantly lower ages and higher MMSE scores than MCI (92.25 ± 2.55VS. 93.26 ± 3.10, P < 0.01 and 26.45 ± 1.45 VS.21.32 ±1.74, P < 0.001 respectively). Subjects without MCI had more exercise habits former than MCI (37/84 VS. 82/265, P < 0.05). Educational level also had significant difference between MCI and normal (illiteracy, 179/213 VS. 34/213; primary school, 89/134 VS. 45/134; secondary school, 7/11 VS 4/11; college and postgraduate, 4/6 VS. 2/6 and P < 0.01). There was no food items and other factors that were different statistically between normal and MCI. Demographic, clinical characteristics of the 364 subjects, grouped by MCI and normal, are shown in Table 1.

**Table 1:** Baseline characteristics according to MCI (n = 364)

	MCI	Normal	χ^2 ^or mean difference
***Gender***			
Men	108/160	52/160	
Women	171/204	33/204	13.35★

***Age, years***	93.26 ± 3.10	92.25 ± 2.55	1.01★
***MMSE scores***	21.32 ± 1.74	26.45 ± 1.45	-5.13★

***Dietary Pattern (times per week)***			
Grain, Cereals	19.61 ± 2.93	19.90 ± 2.78	-0.30
Vegetables	18.13 ± 4.67	18.77 ± 3.99	-0.65
Fruits	2.06 ± 3.18	2.73 ± 3.78	-0.68
Poultry	0.59 ± 1.30	0.81 ± 2.05	-0.22
Pork, Beef, Mutton	5.52 ± 5.77	5.91 ± 6.01	-0.39
Eggs	4.56 ± 4.58	4.27 ± 4.06	0.29
Fish, shrimp	0.21 ± 0.57	0.58 ± 2.45	-0.38
Milk, milk products	1.39 ± 3.20	1.84 ± 3.34	-0.46
legume	2.19 ± 2.58	2.59 ± 3.45	-0.40
Animal oil	6.21 ± 7.06	7.26 ± 8.24	-1.05
Plant oil	16.73 ± 5.76	17.49 ± 5.04	-0.76
Nuts	0.90 ± 2.27	1.68 ± 3.75	-0.78

***Variable***			
SBP (mm Hg)	141.80 ± 22.67	141.23 ± 25.34	0.57
DBP (mm Hg)	73.06 ± 12.55	72.82 ± 11.49	0.24
IBM (Kg/m^2^)	19.55 ± 3.71	19.16 ± 4.13	0.39
FPG (mmol/L)	4.38 ± 1.46	4.62 ± 1.1.58	-0.24
Triglycerides(mmol/L)	1.26 ± 0.72	1.25 ± 0.89	0.01
TC (mmol/L)	4.18 ± 0.81	4.17 ± 0.74	0.01
LDL-C (mmol/L)	2.32 ± 1.34	2.26 ± 0.56	0.06
HDL-C(mmol/L)	1.58 ± 0.44	1.53 ± 0.33	0.04

***Smoking habits***			
Former	170/274	55/84	0.32
Current	121/279	44/85	1.85
***Alcoholic habit***			
Former	120/274	34/83	0.21
Current	76/279	27/85	0.66
***Tea habits***			
Former	133/268	51/83	3.55
Current	133/279	48/85	2.02
***Exercise habits***			
Former	82/265	37/84	4.87▲
Current	110/276	42/85	2.44
***Educational levels***			
Illiteracy	179	34	
Primary school	89	45	
Secondary school	7	4	
College and postgraduate	4	2	15.71★

Abbreviations: MCI, Mild cognitive impairment; MMSE, Mini-Mental State Examination; SBP, systolic blood pressure; DBP, diastolic blood pressure; BMI, body mass index, FPG, fasting plasma glucose; TC, Total cholesterol, HDL-C, high-density lipoprotein cholesterol; LDL-C, low-density lipoprotein cholesterol.

Mean ± s.d.

▲p < 0.05; ★ p < 0.01 vs. mild cognitive impairment group using the χ2 or Fisher's exact test for categorical variables and Independent-Samples t test for continuous variables. P value <0.05 was considered to be statistically significant.

### Dietary Frequencies and Risk of MCI

We assessed whether the 12 dietary items were associated with an increased risk of mild cognitive impairment (Table 2), through comparing the food frequencies in a week. After adjustment for gender, ages, systolic blood pressure, diastolic blood pressure, body mass index, fasting plasma glucose, total cholesterol, triglycerides, high-density lipoprotein cholesterol and low-density lipoprotein cholesterol, smoking habits, alcohol and tea consumption and exercise, there were significant differences revealed between MCI and normal concerning animal oil (ORs, 0.93; 95%CI, 0.88-0.98; P < 0.01) and legume (ORs, 0.84; 95CI%, 0.72-0.97; P < 0.05). As for the other 10 food items, no significant difference was detected in both unadjusted and adjusted models.

**Table 2:** Odds Rations (OR) for Subjects with MCI VS Normal Control by 12 Foods Frequencies in a Week in Continuous.

Food items	Unadjusted (n = 267)	Adjusted (n = 222)
***Grain, Cereals***		
OR(95%CI)	0.99(0.87-1.13)	0.99(0.82-1.21)
***Vegetables***		
OR(95%CI)	0.97(0.89-1.06)	0.96(0.84-1.11)
***Fruits***		
OR(95%CI)	0.95(0.86-1.04)	0.95(0.82-1.09)
***Poultry***		
OR(95%CI)	0.97(0.81-1.15)	1.01(0.77-1.31)
***Mutton, Pork, Beef***		
OR(95%CI)	1.01(0.95-1.07)	1.01(0.92-1.10)
***Eggs***		
OR(95%CI)	1.03(0.96-1.10)	1.05(0.95-1.15)
***Fish, shrimp***		
OR(95%CI)	0.86(0.68-1.08)	0.78(0.40-1.53)
***Milk, milk products***		
OR(95%CI)	0.95(0.87-1.05)	1.01(0.86-1.19)
***Legume***		
OR(95%CI)	0.93(0.84-1.02)	0.84(0.72-0.97)▲
***Animal oil***		
OR(95%CI)	0.96(0.93-1.00)	0.93(0.88-0.98) ★
***Plant oil***		
OR(95%CI)	0.97(0.91-1.03)	0.93(0.85-1.01)
***Nuts***		
OR(95%CI)	0.97(0.88-1.08)	1.07(0.90-1.28)

Binary logistic analyses were used to estimate the OR and 95%CI of 12 food frequencies in a week as a function of increased mild cognitive impairment.

Adjusted for gender, ages, systolic blood pressure, diastolic blood pressure, body mass index, fasting plasma glucose, total cholesterol, triglycerides, high-density lipoprotein cholesterol and low-density lipoprotein cholesterol, smoking habits, alcohol and tea consumption, educational levels and exercise.

▲: P < 0.05

### 12 Food Category Frequencies Distributed among MCI and Normal

**Figure 1:**
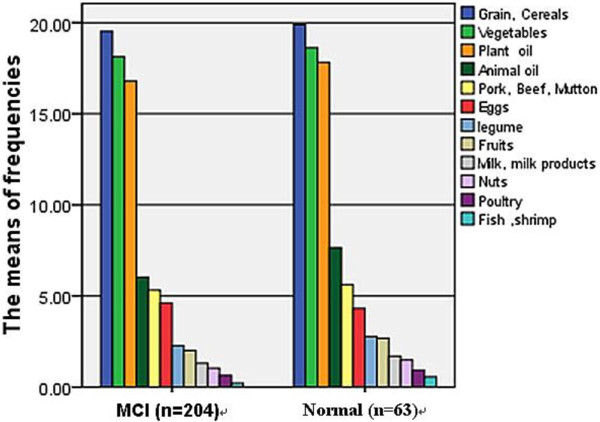
**12 food category frequencies among MCI and normal**.

**Figure 2:**
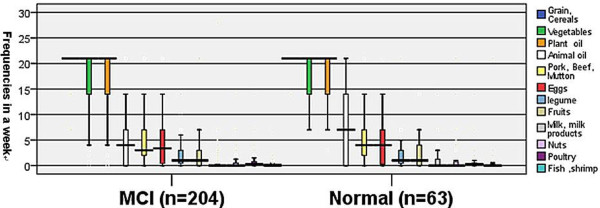
**Box-and-bar plot of 12 food category frequencies in a week in subjects with mild cognitive impairment (MCI) and normal cognitive are shown**.

The means of 12 food category frequencies per week are intuitively showed in figure 1. Subjects in both MCI and normal groups were high intake of grain or cereals, vegetables and plant oil and low intake of animal oil, pork or beef or mutton, eggs, legume, fruits, milk or milk products, nuts, poultry and fish or shrimp. The percentages, the maximal values and the minimum of 12 foods category frequencies in a week were intuitively showed in figure 2. Not much difference was observed for food items between MCI and normal group except animal oil (median was lower than 5 times/week in MCI and higher 5 in normal control; maximum lower than 15 and higher than 20 separately) and milk or milk products (the maximum of normal was nearly to 5 times in a week, but MCI nearly to 0). The frequencies of nuts, poultry and fish or shrimp were paucity, nearly to 0 both in MCI and normal control.

## Discussion

In cross-sectional observation of community-dwelling Chinese nonagenarians and centenarians, there was a high prevalence of mild cognitive impairment, and compared with men, women had a higher prevalence of mild cognitive impairment. Based on cross-sectional observation of community-dwelling oldest persons, we found there were significant associations between the prevalence of mild cognitive impairment and low intake of animal oil and legume, in Chinese nonagenarians and centenarians. But they were affected by other factors, such as gender, ages, former exercise, education, FBG, blood pressure and so on. However, this result of animal oil has some difference with previous cognitive function studies [8,9,13,14], but accord with an Asia study [15]. Animal oil is any oil that obtained from animal substances. Animal oil is not only abundant with saturated fatty acids and Cholesterol, but also much of unsaturated fatty acids (such as fish oil), which is very important for the absorption of fat-soluble vitamins A, D, E, K. In addition, cholesterol in animal oil is an important component of human tissue cells and important raw materials for synthesising bile and some hormones. We can see that both MCI and normal cognitive ones of our participants were mostly vegetarians from figure 1 and figure 2 intuitively and the MCI were malnutrition compared with the normal ones from table 1. To sum up, these findings may prompt that both the too low and the too high levels of animal oil, and will lead to cognitive functions of decline or increase the prevalence of MCI. There are many reports regarding legume protecting cognitive function in the elderly [8,9,15], and in our study, low intake of legume was a risk for MCI, which is in accord with former studies.

This survey showed that there is a high prevalence rate of mild cognitive impairment in Chinese Nonagenarians and Centenarians, more than other large sample surveys [4,16]. The reasons may be as follows: Firstly, although their populations' ages mostly were more than 65-year old, yet the percentage of subjects more than 90 years old was very low. Secondly, our participants mostly lived in Countryside, and their education level were lower (illiteracy 213, non-illiteracy 151, including primary school 134, secondary school 11, college and postgraduate 6). However participants in those surveys had a higher level of education. The illiterate were not as many as ours (213/364). So, there are objective differences between the population in our study and previously.

There were other interesting findings among nonagenarians and centenarians in the present study. Firstly, compared with men, women had a higher prevalence rate of mild cognitive impairment. Secondly, former exercise was related to low prevalence of MCI. Thirdly, educational levels were association with prevalence of MCI, which showed a tendency that the higher education level of participants is, the lower the prevalence of MCI is (illiterates, 84.04%; primary school, 66.42%; secondary school, 63.64%; College and postgraduate, 66.67%).

Our study had some limitations that deserved a mention. Firstly, since it was one part of the PLAD, there might be a survival bias. However, it was inherent in all studies of individuals in this age group. Secondly, because of too many Missing data and to prevent the bias caused by them, we didn't consider the presence depressive symptoms and the number of drugs used. In addition, the conclusion that depression was not directly correlated with cognitive impairment in Chinese nonagenarians and centenarians has been described elsewhere [17]. Thirdly, we did not adjust for other potential confounding factors, such as APOE genotype, socio-economic status and family history of cognitive impairment. Most (90%) participants in the present study lived in the countryside, and some subjects had been working on a farm every day, and so physical activity may be a potential confounder. Thus, this population might not represent the urban population.

## Conclusions

In conclusion, we found there were significant associations between animal oil and legume and the prevalence of MCI among Chinese nonagenarians and centenarians. But it needs to be confirmed by more large studies. No significant associations were detected between the other food items and MCI in this study.

## Competing interests

Prof. Dong serves as the director of Department of Geriatrics West China Hospital, West China School of Medicine, Sichuan University, China. She sponsored the Project of Longevity and Aging in Dujiangyan (PLAD) and was supported by the Discipline Construction Foundation of Sichuan University and by grants from the Project of Science and Technology Bureau of Sichuan Province (2006Z09-006-4), and the Construction Fund for Subjects of West China Hospital of Sichuan University (XK05001).

## Authors' contributions

Ziqi Wang (the first author) drafted the manuscript and performed the statistical analysis. Birong Dong sponsored the Project of Longevity and Aging in Dujiangyan (PLAD) and conceived of the study. Guo Zeng participated in sponsoring PLAD. Jun Li helped to draft the manuscript. Wenlei Wang, Binyou Wang and Qiyuan Quan participated in the questionnaire design and information collected. All authors read and approved the final manuscript.

## Pre-publication history

The pre-publication history for this paper can be accessed here:

http://www.biomedcentral.com/1471-2458/10/595/prepub
